# The tissue distribution and significance of B7-H4 in laryngeal carcinoma

**DOI:** 10.18632/oncotarget.21152

**Published:** 2017-09-21

**Authors:** Lili Chen, Meihua Jin, Chunshi Li, Yongjun Shang, Qinggao Zhang

**Affiliations:** ^1^ Medical College, Dalian University, Dalian, People's Republic of China; ^2^ Department of Clinical Laboratory, Laiwu City People Hospital, Laiwu, People's Republic of China; ^3^ School of Pharmacy, Yanbian University, Yanji, People's Republic of China; ^4^ Department of Orthopedics, Affiliated Hospital of Chifeng University, Chifeng, People's Republic of China

**Keywords:** B7-H4, laryngeal carcinoma, invasion and metastasis, EMT, STAT3

## Abstract

The costimulatory signals CD28 and B7 have been shown to control tumor invasion and metastasis by regulating T cell activation, whereas the distribution characteristics of B7-associated proteins in laryngeal carcinoma (LC) tissue are still unclear. Here, the expression of members of the B7 superfamily, including B7-H1 (PD-L1), B7-DC (PD-L2) and B7-H4, in fifty-two LC samples was determined by immunohistochemistry, and the relationship between B7-H4 and epithelial-mesenchymal transition (EMT)-associated markers was further assessed by immunofluorescence double staining. Furthermore, the human LC cell lines, Hep-2 and TU212 cells, were further transfected to overexpress B7-H4, and cell invasion and metastasis were analyzed. The results showed that B7-H1, B7-DC and B7-H4 were expressed in the tumor cells, and their expression was restricted to the cell membrane and the cytoplasm. The positive rates of these molecules in the tumor tissues were 57.7% (30/52), 32.7% (17/52) and 34.6% (18/52), respectively. Interestingly, double immunofluorescence staining showed that B7-H4 is coexpression with EMT-related markers, including p-Smad2/3, Snail and Vimentin, in carcinoma cells. Moreover, overexpression of B7-H4 in Hep-2 cells promotes the expression of pSmad2/3 and Snail by activating AKT-STAT3 signaling. Transwell and wound-healing assays demonstrated that B7-H4 enhanced both Hep-2 and TU212 cell invasion and metastasis. Our results suggest that B7-H4 transmits feedback signaling to tumor cells and promotes invasion and metastasis by promoting EMT progression. Therefore, blocking B7-H4 signaling might be a novel treatment strategy for LC.

## INTRODUCTION

Laryngeal carcinoma (LC), which is derived from epithelial cells (the most common squamous cell) and accounts for 0.8% of all new cancer cases, is the 14^th^ most common cancer among men [1]. According to GLOBOCAN, LC caused 83376 deaths in 2012 (3880 in the USA and 12308 in China), and males are much more susceptible to LC than females (10550 new cases in males compared to 2880 females in 2015) [1]. Etiological studies report that smoking and alcohol consumption are the major causes. Moreover, exposure to several other environmental factors, such as asbestos, polycyclic aromatic hydrocarbons, and textile dust, is thought to potentially increase the risks [2]. The development of LC is also racial biased, with African Americans presenting higher incidence rates and mortality compared with Caucasians [3]. Significant advancements have been made over the past several decades in the treatment of LC. Surgery is an integral part of treatment, while nonsurgical approaches such as radiation and systemic therapy have emerged as viable options. Unfortunately, the 5-year survival rate of LC patients has decreased from 66% to 63% over the past 40 years, even though the overall incidence has declined [4]. It is critical to explore the pathogenic and prognostic indicators of LC due to the fact that the molecular tests have not influenced LC treatment selection until now.

Immunotherapy has recently emerged as the fourth pillar of cancer treatment, joining surgery, radiation, and chemotherapy. Immune checkpoint inhibitors, such as the monoclonal antibodies (mAb) that target cytotoxic T lymphocyte-associated antigen-4 (CTLA-4) and programmed death-1 (PD-1), are at the forefront of immunotherapy development [5]. CTLA-4 and PD-1, two members of the CD28 costimulatory receptor superfamily, transmit inhibitory signals that prevent antigen-mediated T cell activation and thus terminate immune responses. As ligands of PD-1, B7-H1 (PD-L1) could be expressed in many types of cells, including tumor cells, immune cells, epithelial cells, and endothelial cells, whereas B7-DC (PD-L2) is primarily expressed in antigen-presenting cells (APCs), such as macrophages and DCs [6]. For example, the expression levels of B7-H1 were significantly higher in poorly and moderately differentiated LCs compared to the levels in well-differentiated patients [7]. Additionally, increased tumor-infiltrating lymphocyte (TIL) density and B7-H1 levels are associated with better outcomes in LC patients [8]. B7-H4, also known as B7x or B7S1, is a recently identified co-inhibitor of the B7 superfamily that can inhibit proliferation, activation and cytokine production in T cells by interacting with an unidentified receptor [9]. B7-H4 is expressed in many tumor cells, including ovarian cancer cells, gallbladder carcinoma cells, renal cell carcinoma cells and hepatitis B virus-related hepatocellular carcinoma cells [10–12]. Nevertheless, the expression and distribution of B7-H4 in LC samples is unreported.

The concept of epithelial-mesenchymal transition (EMT) was first proposed by Greenburg in 1982 [13]. EMT is a biological process in which epithelial cells lose their original polarity in certain physiological or pathological conditions, becoming mesenchymal cells that can move freely in the cell matrix. This has been reported to play an important role in organ fibrosis, tumor invasion and tumor metastasis [14]. Interestingly, the expression of the EMT-related marker Snail is positively correlated with LC recurrence after surgery [15]. The positive expression rate of Snail mRNA is higher in LC tissue than in the laryngeal chronic inflammatory and atypical hyperplasia tissues of the throat, and Snail mRNA is associated with the pathology grade, TNM stage, distant metastasis and lymphatic metastasis of LC [16]. These studies implicate that EMT can enhance the metastatic ability of LC.

In this research, we investigated the expression and distribution of three members of the B7 superfamily, B7-H1, B7-DC and B7-H4, in fifty-two LC samples and ten peritumoral normal tissues by immunohistochemistry. Moreover, we further analyzed the potential role of B7-H4 in regulating tumor cell invasion and metastasis.

## RESULTS

### The expression and distribution of B7-H1, B7-DC and B7-H4 in laryngeal carcinoma samples

**Figure 1:**
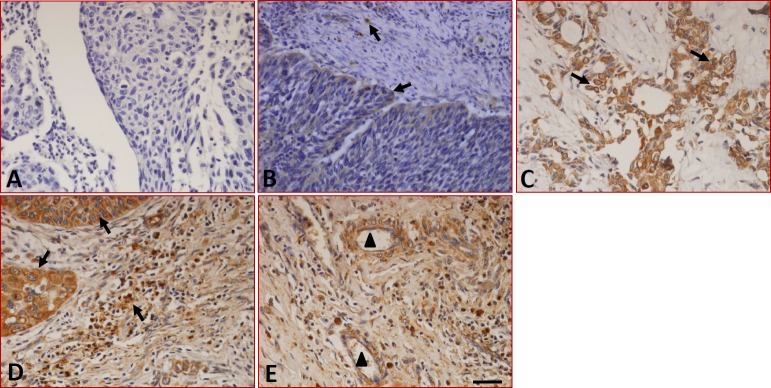
Expression of B7-H1 in LC tissues detected by immunohistochemistry **(A)** Sections were stained with isotype-specific mouse IgG1 control antibodies. **(B)** Low levels of B7-H1 were seen in the infiltrated T cells and epithelial cells of peritumoral normal tissues. **(C)** B7-H1 was expressed on the surface and in the cytoplasm of the tumor cells, **(D)** tissue-infiltrating lymphocytes and **(E)** the blood capillaries of tumor tissues. The arrows indicate positive cells. Scale bar = 20 μm, ▲ indicated blood capillaries.

We first detected the expression and distribution of B7-H1 in fifty-two LC samples and ten cases of peritumoral normal tissues by immunohistochemistry. The results showed that although samples stained with isotype-specific mouse IgG antibodies were negative for B7-H1 (Figure 1A), low levels of B7-H1 were seen in the epithelial cells and infiltrated monocytes (Figure 1B) of the peritumoral normal tissues. Conversely, significantly higher levels of B7-H1 were found in the tumor cells (Figure 1C), tissue-infiltrating lymphocytes (Figure 1D), blood capillaries (Figure 1E) of tumor tissues, and the expression was found to be restricted to the cell surface and the cytoplasm. 57.6% (30/52) of the tumor samples were positive for B7-H1 in the tumor cells. The expression and distribution characteristics of B7-H1 in these tumor samples are shown in Table 1.

**Table 1:** The expression of B7 family members including B7-H1, B7-DC and B7-H4 in human laryngeal carcinoma

Case numb.	B7-H1	B7-DC	B7-H4	Tumor recurrence
1	Carcinoma cells(+)	-	Carcinoma cell (++), lymphocytes(+), capillaries (+)	Yes
2	capillaries (++)	-	Capillaries(+)	No
3	Carcinoma cells (++++), capillaries (+), lymphocytes (++)	-	Lymphocytes(++)	No
4	Carcinoma cells (+++), capillaries (++), lymphocytes (++)	-	Carcinoma cells (++)	No
5	lymphocytes(+)	-	-	No
6	lymphocytes (++++), capillaries (+),	-	Carcinoma cells (++)	No
7	lymphocytes (+)	Carcinoma cells (++)	-	No
8	Carcinoma cells (++), capillaries (+)	-	-	No
9	Carcinoma cells (++++), lymphocytes (++++), capillaries (+++)	-	Carcinoma cells (++++), capillaries (+++), lymphocytes (+++)	No
10	Carcinoma cells (+++), lymphocytes (+)	-	Carcinoma cells (+), capillaries (+)	No
11	Carcinoma cells (++), capillaries (+)	-	capillaries (++)	No
12	capillaries (+++), Carcinoma cells (++++), lymphocytes (++)	-	lymphocytes (++++), Carcinoma cells (++++)	No
13	Carcinoma cells (++++), capillaries (+++)	-	capillaries (++)	No
14	lymphocytes (+)	-	-	No
15	Carcinoma cells (++)	-	lymphocytes (+++), capillaries (+)	No
16	Carcinoma cells (+)	-	Epithelial cells (+)	No
17	Carcinoma cells (+)	-	Epithelial cells (++)	No
18	Epithelial cells (++), lymphocytes (+++)	Epithelial cells (+++), lymphocytes (++)	lymphocytes (+++), Epithelial cells (++)	No
19	Carcinoma cells (++++), capillaries (++++)	Carcinoma cells (++++), capillaries (++++)	Carcinoma cells (++++), lymphocytes (++)	No
20	Carcinoma cells (++++), lymphocytes (++++), capillaries (++)	Carcinoma cells (+++), lymphocytes (++++), capillaries (+++)	Carcinoma cells (+), lymphocytes (+++)	No
21	-	-	lymphocytes (+)	No
22	lymphocytes (+)	-	lymphocytes (+)	No
23	capillaries (+)	capillaries (+)	lymphocytes (+++), capillaries (-)	No
24	Carcinoma cells (++++), epithelial cells (+), capillaries (++), lymphocytes (++)	Carcinoma cells (++++), lymphocytes (+)	Carcinoma cells (++), lymphocytes (++++)	No
25	Carcinoma cells (++++)	Carcinoma cells (++++)	-	No
26	lymphocytes (++), epithelial cells (+)	Carcinoma cells (++++), lymphocytes (+), epithelial cells (+)	Carcinoma cells (+), lymphocytes (++)	No
27	epithelial cells (+++), Carcinoma cells (+)	Carcinoma cells (+++), epithelial cells (++)	capillaries (+)	No
28	Macrophages (++)	Carcinoma cells (+++), lymphocytes (+++)	epithelial cells (++), capillaries (+++), Carcinoma cells (+)	No
29	Carcinoma cells (+)	Carcinoma cells (+-)	-	No
30	Carcinoma cells (++++), lymphocytes (+++), capillaries (+++)	-	lymphocytes (++++), Carcinoma cells (+)	No
31	Carcinoma cells (++++), lymphocytes (+++), capillaries (+++)	Carcinoma cells (++), capillaries (++)	Carcinoma cells (+++), lymphocytes (+++)	No
32	capillaries (+)	capillaries (+)	capillaries (+)	No
33	capillaries (++)	Carcinoma cells (++)	Carcinoma cells (+-), lymphocytes (+++)	No
34	Carcinoma cells (+)	-	-	No
35	-	Carcinoma cells (++)	-	No
36	Carcinoma cells (++)	-	Carcinoma cells (+), lymphocytes (+)	No
37	Carcinoma cells (++), lymphocytes (++)	-	Carcinoma cells (++)	Yes
38	Carcinoma cells (+++)	-	-	No
39	capillaries (+++), Carcinoma cells (+)	-	lymphocytes (+++)	No
40	Carcinoma cells (+++)	lymphocytes (+)	lymphocytes (++)	No
41	-	Carcinoma cells (+)	capillaries (++),	No
42	lymphocytes (++), Carcinoma cells (++)	Carcinoma cells (+-)	-	No
43	lymphocytes (++), Carcinoma cells (+)	lymphocytes (++)	lymphocytes (+)	No
44	Carcinoma cells (+++), lymphocytes (+)	Carcinoma cells (+++), lymphocytes (+)	lymphocytes (+++), Carcinoma cells (+)	No
45	Carcinoma cells (++++)	Carcinoma cells (++++)	Carcinoma cells (+), capillaries (+)	No
46	Carcinoma cells (++)	Carcinoma cells (++)	Carcinoma cells (+), lymphocytes (+++)	No
47	-	-	Carcinoma cells (++), lymphocytes (+)	Yes
48	Carcinoma cells (+)	Carcinoma cells (+)	Carcinoma cells (+)	Yes
49	-	-	-	No
50	-	Lymphocytes (++)	-	No
51	Carcinoma cells (+), lymphocytes (+)	Carcinoma cells (++), lymphocytes (+++)	Lymphocytes (+++)	No
52	Carcinoma cells (+)	lymphocytes (++)	lymphocytes (++)	No

−: negative; +: weak positive; ++: meddle positive; +++: strong positive.

**Figure 2:**
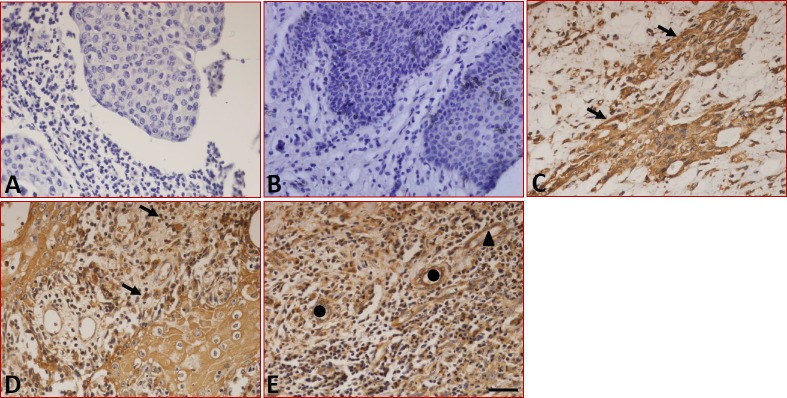
Expression of B7-DC in LC tissues detected by immunohistochemistry **(A)** Sections were stained with isotype-specific mouse IgG1 control antibodies. **(B)** B7-DC was absent in peritumoral normal tissues. B7-DC was expressed on the surface and in the cytoplasm of tumor cells **(C),** tissue-infiltrating lymphocytes **(D)**, blood capillaries and epithelial cells **(E)**. Arrows indicate positive cells, ▲ indicates blood capillaries and ● indicates epithelial cells. Scale bar = 20 μm.

The sections stained with the isotype-specific mouse IgG antibodies and the peritumoral normal tissues were negative for B7-DC (Figure 2A and 2B). In tumor tissues, immunohistochemistry showed that B7-DC was expressed in the tumor cells (Figure 2C), tissue-infiltrating lymphocytes and blood capillaries (Figure 2D), moreover, B7-DC was also found in epithelial cells (Figure 2E). Similar to B7-H1, B7-DC expression was restricted to the cell surface and the cytoplasm. The expression and distribution characteristics of B7-DC in the fifty-two samples are shown in Table 1, and 32.7% (17/52) of the tumor cell samples were positive for B7-DC in tumor cells.

**Figure 3:**
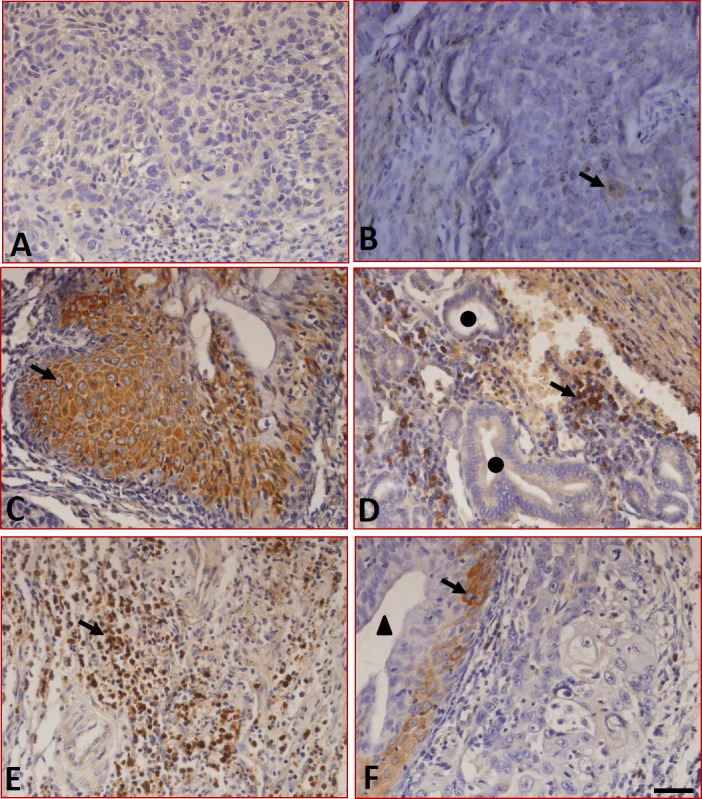
Expression of B7-H4 in LC tissues detected by immunohistochemistry **(A)** Sections were stained with isotype-specific mouse IgG1 control antibodies. **(B)** B7-H4 was absent in peritumoral normal tissues. In tumor tissues, B7-H4 was expressed on the surface and in the cytoplasm of tumor cells **(C)** and in tissue-infiltrating macrophages **(D)**, and tissue-infiltrating lymphocytes **(E)**, whereas it was absent in the blood capillaries **(F)**. ▲ indicates blood capillaries and ● indicates epithelial cells. Arrows indicate positive cells. Scale bar = 20 μm.

We further analyzed the expression of B7-H4 in the LC samples using immunohistochemistry. The results showed that although sections stained with isotype-specific mouse IgG antibodies were negative for B7-H4 (Figure 3A), sections from peritumoral tissues were positive for B7-H4 and positive cells are macrophages/monocytes (Figure 3B). In tumor tissues, the expression of B7-H4 was not only found in carcinoma cells (Figure 3C) but also in the tissue-infiltrating lymphocytes (Figure 3D) and macrophages (Figure 3E), but B7-H4 was absent from the capillaries (Figure 3F). Additionally, the expression of B7-H4 was restricted to the cell surface and the cytoplasm, 34.6% (18/52) of the tumor samples were positive for B7-H4 in tumor cells, and the expression and distribution characteristics of B7-H4 are shown in Table 1.

We also analyzed the expression and clinical pathological features of these molecules, including survival time and tumor recurrence. Interestingly, until now, no patients had died after surgery, and only three patients experienced tumor recurrence. B7-H4 was highly expressed in the tumors of all three of these patients, but B7-H1 and B7-DC were negatively or slightly expressed (Table 1), suggesting that **B7-H4 might promote tumor recurrence**. Previous work has shown that B7-H1 is expressed in human LC tissues, and its expression could be useful to predict responses to immune checkpoint inhibitors [17]. Therefore, we selected to further study B7-H4.

### B7-H4 is coexpressed with EMT-associated markers in laryngeal carcinoma cells

**Figure 4:**
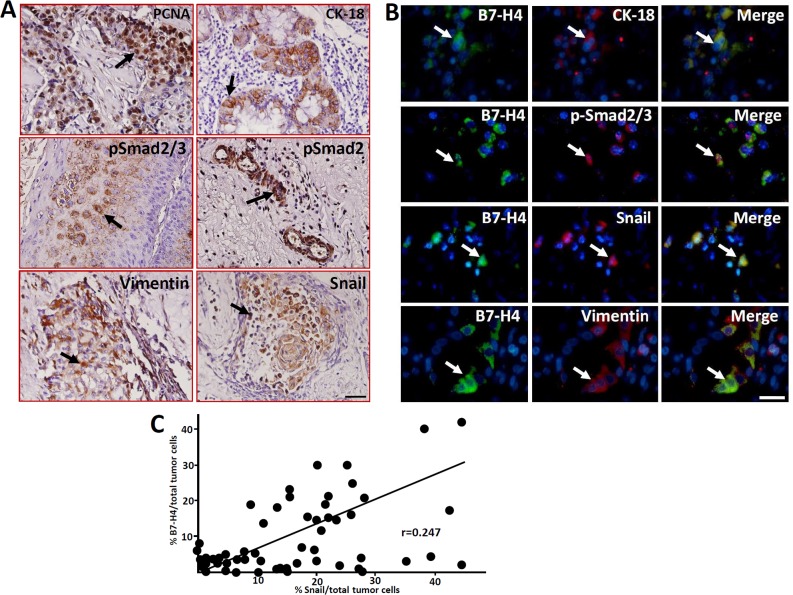
Expression of the cell proliferation marker PCNA and EMT-related markers in human LC samples detected by immunohistochemistry **(A)** The expression of PCNA, CK18, p-Smad2/3, p-Smad3, Vimentin and Snail in laryngeal carcinoma cells was detected by immunohistochemistry. Arrows indicate positive cells. Scale bar = 20 μm. **(B)** The co-expression of the EMT-related markers and B7-H4 in LC cells was detected by immunofluorescence double staining. The arrows indicate positive cells and DAPI indicates nuclear staining. Scale bar = 20 μm. **(C)** Statistical analyzed the relationship of %B7-H4positive and % Snail positive cells in total tumor cells of these fifty-two LC samples, r=0.247.

EMT is involved in a variety of physiological and pathological process, such as embryogenesis, organ development, tissue repair, organ fibrosis, tumor invasion and metastasis [18, 19]. Immunohistochemistry was performed to detect the expression of EMT-related markers in the LC samples. As shown in Figure 4A, the EMT-associated markers CK-18, p-Smad2/3, p-Smad3, Snail and Vimentin, as well as the cell proliferation marker PCNA, were expressed in the LC samples. Double immunofluorescence staining showed that B7-H4 was co-expressed with CK-18, p-Smad2/3, Snail and Vimentin (Figure 4B). These data suggest that B7-H4 might promote the pathogenesis of LC by inducing EMT progression.

We also analyzed the relationship between B7-H4 and Snail in the LC samples because the expression of Snail has been suggested to positively correlate with LC recurrence after surgery [15, 16]. Interestingly, the expression of B7-H4 was positively correlated with Snail in the tumor cells (r=0.247, Figure 4C). These data suggest that B7-H4 might promote the pathogenesis of LC by inducing EMT progression.

### Overexpression of B7-H4 in Hep-2/TU212 cells promotes the expression of EMT-associated markers p-Smad2/3 and Snail

**Figure 5:**
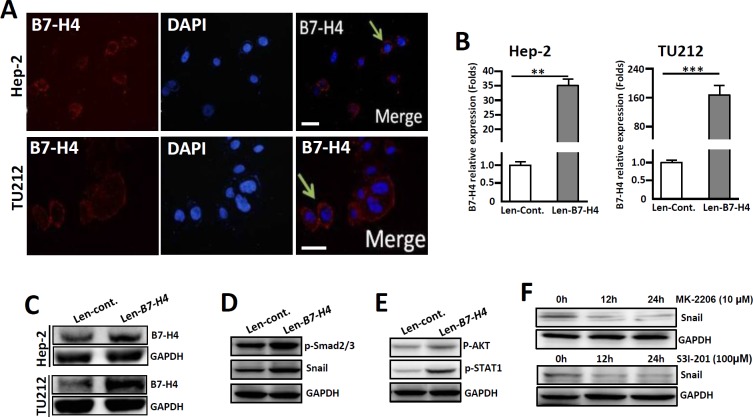
Overexpression of B7-H4 in both Hep-2 and TU212 cells promotes the expression of EMT-associated markers **(A)** Immunofluorescence shows endogenous expression of B7-H4 in Hep-2/TU212 cells. The arrow indicates positive cells. Blue, DAPI; Red, B7-H4. Scale bar =20 μm. **(B)** qRT-PCR was used to detect B7-H4 in Hep-2/TU212 cells by lentiviral infection (*Len-B7-H4*) and control (Len-*Cont*.). **(C)** B7-H4 protein expression in Hep-2/TU212 cells was measured by western blot. **(D)** The expression of EMT-associated markers, including p-Smad2/3 and Snail, in Hep-2 cells in which B7-H4 was overexpressed and the control counterparts was compared by western blot. **(E)** The expression of p-AKT and p-STAT-1 in Hep-2 cells in which B7-H4 was overexpressed and the control counterparts was compared by western blot. **(F)** Hep-2 cells in which B7-H4 was overexpressed were treated with the Akt inhibitor MK-2206 or the STAT-1 inhibitor S3I-201 for 0 h, 12 h and 24 h, and the expression of Snail was detected by western blot. One of three experiments that had comparable results is shown.

The human LC cell line Hep-2 was used for *in vitro* studies on the roles of B7-H4 in regulating tumor cell invasion and metastasis. Immunofluorescence staining showed that both Hep-2 and TU212 cells have slight endogenous B7-H4 expression levels (Figure 5A). However, cells transfected with lentiviral-mediated B7-H4 (Len-*B7-H4*) resulted in an overexpression of B7-H4 compared to its control counterpart (Len-*Cont.*), as detected by qRT-PCR and Western blot, respectively (Figure 5B and 5C)

To analyze whether B7-H4 would promote the progression of EMT *in vitro*, the expression levels of the EMT-associated markers p-Smad2/3 and Snail in Hep-2 cells were compared. Western- blot analysis showed that Hep-2 cells in which B7-H4 was overexpressed had higher levels of p-Smad2/3 and Snail compared to those of the cells that were transfected with the Len-*cont.* counterpart (Figure 5D). Additionally, the expression of p-AKT, and subsequently, p-STAT1, was also upregulated in B7-H4-overexpressed Hep-2 cells (Figure 5E). Conversely, an interruption of AKT phosphorylation by the Akt inhibitor MK-2206 or inhibition of STAT-1 phosphorylation by the inhibitor S3I-201 leads to the downregulation of Snail expression in Len-*B7-H4*-transfected Hep-2 cells (Figure 5F). These combined data suggest that B7-H4 promotes EMT-associated molecule expression in Hep-2 cells by inducing AKT/STAT1 signaling.

**Figure 6:**
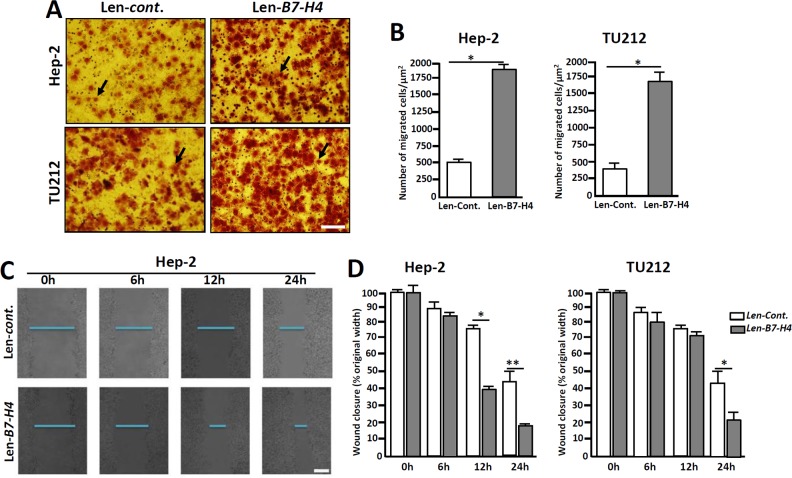
Overexpression of B7-H4 in Hep-2/TU212 cells promotes cell invasion and metastasis **(A)** Representative images of cell invasion by transwell chamber assay. **(B)** Cell invasion assays of Hep-2/TU212 cells with or without B7-H4 overexpression. The results are the mean ± SEM (standard error of the mean) from three independent experiments. ^*^ *p*<0.05. **(C)** and **(D)** Cell migration by wound healing analysis. The confluent cells were wounded by sterile pipettes and the status of wound closure were observed and photographed after 6 h, 12 h and 24 h of culture. All the experiments were repeated for three times. Scale bar=20μm. ^*^ *p*<0.05 and ^**^ *p*<0.01.

The invasion and metastatic abilities of Hep-2/TU212 cells were further analyzed. Similar to the changes observed in the EMT-related markers, highly expressed B7-H4 in Hep-2 and TU212 cells induced increased cell migration by approximately 3.5- and 4.13- fold respectively, as compared to that of the control counterparts (Figure 6A and 6B). Furthermore, wound healing experiments showed that overexpression of B7-H4 in Hep-2 cells led to a higher migration rate compared to that of the controls, especially at 24 h (Figure 6C). These combined data suggest that B7-H4 promotes tumor cell invasion and metastasis *in vitro*.

## DISCUSSION

In addition to the expression of APCs, the costimulatory B7-related proteins have been shown to be expressed in many types of cancer tissues, and they could provide positive or negative signals to control the local T cells that control cancer development [20]. Selective enhancements of T cell activation using PD-1 or CTLA-4 blocking antibodies have been demonstrated to be a suitable strategy for cancer immunotherapy [21]. Recently, many costimulatory molecules of the B7 superfamily have been identified. Understanding their expression and distribution characteristics is essential for the development of novel treatment approaches for carcinoma [22]. Here, we analyzed the expression of B7-H1, B7-DC and B7-H4 in fifty-two LC samples and ten peritumoral normal tissues by immunohistochemistry. The results showed that B7-H1, B7-DC and B7-H4 are expressed in tumor cells, and the expression is restricted at the cell membrane and in the cytoplasm. The positive rates of these molecules in tumor cells were 57.7% (30/52), 32.7% (17/52) and 34.6% (18/52), respectively. Double immunofluorescence staining further showed that B7-H4 is coexpressed with EMT-related markers, such as p-Smad2/3, Snail and Vimentin, in carcinoma cells. Additionally, overexpressed B7-H4 enhanced the invasion and metastasis of Hep-2 cells, an LC cell line, probably through the promotion of EMT progression. These results suggest that B7-H4 could reverse the signal to tumor cells and promote cell invasion and metastasis. Therefore, blocking B7-H4 signaling might be a novel treatment strategy for LC.

B7-H4 is a co-inhibitory molecule of the B7 family that can inhibit T cell activation and proliferation, as well as cytokine secretion [23]. The expression of B7-H4 has been reported in many types of carcinoma. For example, B7-H4 is expressed in colon and gastric cancer, and the expression is related to the tumor invasion depth and lymph node metastasis [24]. An overexpression of B7-H4 can also significantly shorten the lifespan of gastric cancer patients [25]. In addition, B7-H4 can reduce cell-cell adhesion and increase the formation of pseudopodia in pancreatic cancer cells [26, 27]. *In vitro*, B7-H4 enhances oncogenicity and inhibits apoptosis in pancreatic cancer cells [28]. It has also been reported that the expression of B7-H4 is associated with TNM staging and the pathological grading of bladder cancer and thyroid cancer [29, 30]. Additionally, soluble B7-H4 can be used as a diagnostic and prognostic marker of malignant pleural effusion [31]. However, the expression and the potential role of B7-H4 in LC development have not been reported. Here, we found that B7-H4 was not only expressed in carcinoma cells but also in tissue-infiltrating lymphocytes and macrophages. However, peritumoral tissues were negative for B7-H4 (Figure 3). Of the tumor samples, 34.6% (18/52) were positive for B7-H4, and interestingly, 3 of the patients experienced tumor recurrence. All 3 of these patients had highly expressed B7-H4 (Table 1), suggesting **that B7-H4 might promote tumor recurrence**.

It has been shown that the TGF-β/TGFR interaction leads to the phosphorylation of Smad2/3, which causes the upregulation of EMT-associated markers, including Snail, N-cadherin and Twist, by increasing the expression of ZEB and other transcription factors [32, 33]. CK-18 is an intermediate filament protein that is the most reliable marker for epithelial differentiation [34]. Here, we showed that CK-18 is expressed in human LC tissues (Figure 4A), suggesting that human LC originates from epithelial cells. Additionally, the EMT-related markers, including p-Smad2/3, p-Smad3, Snail and Vimentin, as well as the cell proliferation marker PCNA, were expressed in the LC samples (Figure 4A). The relationships between B7 proteins and EMT in tumor development have been researched, and previous work has shown that the upregulation of B7-H1 in skin epithelial cells accelerates carcinogenesis by promoting EMT [35]. Moreover, it was shown that RCC-associated B7-H1 can induce EMT and enhance RCC cell cancer stemness by upregulating SREBP-1c [36]. Additionally, B7-H3 also promotes EMT in colorectal cancer [37]. Recent work has shown that B7-H3 promotes the aggression and invasion of hepatocellular carcinoma by targeting EMT transition *via* the JAK2/STAT3/Slug signaling pathway [38]. Here, we showed that B7-H4 is coexpression with EMT-associated markers (Figure 4B), suggest that B7-H4 might promote the pathogenesis of LC through inducing EMT progression. To confirm this possibility, Hep-2 and TU212 cells were transfected to overexpress B7-H4, and the results showed that the invasion and metastatic abilities of B7-H4-transfected Hep-2 cells were significantly higher than those of the control counterparts (Figure 6), indicating B7-H4 accelerates the invasion and metastasis of LC.

LC remains one of the most common tumors of the respiratory tract. Although surgery has been the historical mainstay for treating localized disease and remains an integral part of treatment, nonsurgical options, such as radiation and systemic therapy, have emerged as viable options [39]. Immune checkpoints, such as PD-1, are manipulated by tumors to allow tumor growth. Overexpression of PD-1 ligands, including PD-L1 and PD-L2, by tumor cells could activate the PD-1 checkpoint pathway, thus leading to an attenuated immune response [40]. Therefore, antibodies targeting the immune PD-1 checkpoint could “release the brakes” and induce an immune response against tumors [41]. Here, we found that B7-H1, B7-DC and B7-DC were expressed in the LC samples. Conversely, the expression of B7-H4 was absent in the capillaries and the peritumoral normal tissues, but these tissues expressed B7-H1 and B7-DC (Figure 3). Together, these data suggest that **B7-H4 might be a more specific immune checkpoint** for LC immunotherapy than B7-H1 and B7-DC.

Taken together, our results demonstrate that the B7 family members B7-H1, B7-DC and B7-H4 are expressed in human LC tissues. All the members, but especially B7-H4, might be involved in the invasion and metastasis of LC by promoting the EMT process. Thus, the possibility of using B7-H4 as a therapeutic target could be explored.

## MATERIALS AND METHODS

### Laryngeal carcinoma cell lines

Hep-2 cells were purchased from Shanghai Bogoo Biotechnology, TU212 cells were purchased from ATCC. Cells were grown in RPMI-1640 culture medium containing 10% fetal bovine serum at 37°C in a humidified incubator with 5% CO_2_.

### Laryngeal carcinoma samples and peritumoral normal tissues

Fifty-two LC samples and ten peritumoral normal tissues were provided by the E.N.T. department at Xinqiao Hospital affiliated with Third Military Medical University. The samples were fixed with 4% polyformaldehyde and embedded in paraffin wax prior to sectioning.

### Immunohistochemistry

The expression of the B7 proteins and the EMT-associated markers was examined in serial sections of LC samples. IHC was performed on the selected slides as previously described [42]. Briefly, paraffin-embedded tissue blocks were cut into 2.5 μm sections and mounted on poly-L-lysine-charged glass slides. After the sections were dewaxed and rehydrated, antigen retrieval was performed by adding 10 mM citrate buffer (pH 6.0) and microwaving. The sections were cooled to room temperature (RT), and the endogenous peroxidase activity was blocked by incubating the sections with a solution of 0.5% H_2_O_2_ in 50% methanol for 30 min. The sections were then incubated in 2% BSA for 1 h at RT to block nonspecific binding. Then, the sections were incubated overnight at 4°C with primary antibodies: anti-B7-H1 (1:150, Clone: #130021, R&D Systems, Minneapolis, MN, USA), anti-B7-DC (1:100, Clone: #176611, R&D Systems), anti-B7-H4 (1:100, Clone: #935317, R&D Systems), anti-CK-18 (1:50, Santa Cruz, San Diego, CA, USA), anti-PCNA (1:50, Santa Cruz), anti-Vimentin (1:50, Santa Cruz), anti-Snail (1:50, Abcam, San Diego, CA, USA), and anti-pSmad2/3, (1:100, Abcam). After washing, the sections were incubated with the corresponding secondary antibodies for 2 h at RT. The Vecta-stain ABC kit (Vector Laboratories, San Diego, CA, USA) was used to perform the avidin–biotin complex method according to the manufacturer's instructions. Sections incubated with isotype- and concentration-matched immunoglobulins without primary antibodies were used as isotype controls. Peroxidase activity was visualized with the DAB Elite kit (K3465, DAKO), and brown coloration of tissues represented positive staining. The sections were lightly counterstained with hematoxylin, dehydrated to xylene through an ethanol series and mounted. Finally, sample sections were viewed using a light microscope (Zeiss Axioplan 2, Berlin, Germany).

### Immunofluorescence double staining

Sections were incubated with primary anti-B7-H4 antibodies at 4°C overnight. After washing with PBS (3 washes, 5 min per wash), the sections were incubated with Alexa Fluor^555^-conjugated goat anti-mouse IgG antibodies (Invitrogen, San Diego, CA, USA) for 1 h. The sections were further incubated with anti-CK18, anti-Vimentin, anti-Snail, or anti-pSmad2/3 antibodies at 4°C overnight and incubated with Alexa Fluor^488^-conjugated goat anti-mouse/rabbit IgG1 antibodies (Invitrogen) for an additional 1 h. Finally, the sections were incubated with 1 lμg/ml DAPI (Sigma, St. Louis, MO, USA) for 10 min to stain the nuclei. Sections incubated with the appropriate isotype-specific control primary antibodies and fluorescently labeled secondary antibodies were used as negative controls. The results were analyzed using fluorescence microscopy (Zeiss Axioplan 2).

### Lentiviral constructs and transduction

The human *B7-H4* (NM_024626.3) cDNA ORF clone (#RC210360) was purchased from OriGene Technologies, Inc. (Rockville, MD, USA). The cDNA for B7-H4 (the whole gene) was further amplified with the following specific primers: Sense: 5′-AGTCAGATCTCCACCATGTTCAGAGGCCGGACAGC-3′; Antisense: 5′-AGTCGAATT CTTATTTTAGCATCAGGTAAG-3′. The cDNA was cloned into the MigR1V1 vector. The lentiviral packaging vectors psPAX2 and pVSVG were purchased from Addgene (Cambridge, MA, USA). The psPAX2 plasmids (2 μg), the expression vectors (2 μg) and the pVSVG plasmids (2 μg) were cotransfected into 293T cells, and the virus supernatants were collected after 48 h (2000 rpm/min, 3 min). Hep-2 and TU212 cells (2×10^5^/well) were seeded in 6-well plates 24 h prior to transfection. When the cells in each well reached approximately 50% confluency, they were transduced with unconcentrated virus supernatant overnight in the presence of 8 mg/ml polybrene and selected in puromycin (0.5 mg/ml). The B7-H4 mRNA levels were evaluated by quantitative polymerase chain reaction (q-PCR), and the cell protein levels were evaluated by Western-blotting.

### Real time RT-PCR

Total RNA from Hep-2/TU212 cells was prepared using an RNeasy Kit according to the manufacturer's instructions. qRT-PCR was carried out using SYBR Premix Ex *Taq* (Takara) according to the manufacturer's instructions with a 20 ng template and the following primers: B7-H4 forward: 5′-ACTCAC AGATGCTGGCACCTAC-3; reverse: 5′-TTGGCTCCCT GGTCAACTTGG-3′; β-actin forward: 5′-CACTATCGGC AATGAGCGGTTCC-3′; reverse: 5′-CAGCACTGTGTT GGCATAGAGGTC-3′. The results were compared using the 2*^−ΔΔCt^* method.

### Western blot analysis

The cells were lysed using cell lysis buffer, and Western- blotting was performed. Briefly, the cell lysates were denatured for 10 min at 95°C with SDS-polyacrylamide gel electrophoresis (SDS-PAGE) sample buffer, electrophoresed on 10% SDS-PAGE gels and transferred to polyvinylidene difluoride membranes. The membranes were blocked with 5% nonfat milk in TBST [20 mM Tris-HCl (pH 7.5), 150 mM NaCl, 0.05% [v/v] Tween 20] and then incubated with specific B7-H4 antibodies (1:500, R&D Systems) for 2 h at RT. After washing, the blots were incubated with HRP-conjugated secondary antibodies. Protein band intensities were analyzed using a chemical color analyzer (Kodak Image Station 4000 MM, Kodak Molecular Imaging System, New Haven, CT, USA).

### Transwell invasion assay

The cell migration assay was performed as described previously [13]. Briefly, after being digested with 0.25% trypsin, Hep-2/TU212 cells were quantified by the cell counting plate method under an optical microscope. Then, 4 × 10^4^ cells were seeded in serum-free medium in the insert, and the lower chamber was filled with 10 % FBS + DMEM medium. After 24 h in a humidified incubator with 5% CO_2_ at 37°C, the inserts were stained with crystal violet, and the invading cells were counted for at least five different fields.

### Wounded-healing assays

Hep-2 cells were incubated for 48 h. Cell monolayers were then damaged using a 10 μl plastic tip, and cell migration into the wounded area was measured after 0 h, 6 h, 12 h and 24 h.

### Statistical analysis

All statistical analysis was carried out using GraphPad Prism 5.0 software. Data are presented as the means ± SEM. Analysis was performed using the paired or unpaired Student's t test as appropriate. For all experiments, statistical significance was accepted at p<0.05.

## Abbreviations

EMT: epithelial mesenchymal transition
APC: antigen presentation cells
